# Split-filter dual energy computed tomography radiotherapy: From calibration to image guidance

**DOI:** 10.1016/j.phro.2023.100495

**Published:** 2023-09-25

**Authors:** Jens Edmund, Marianne Feen Rønjom, Mette van Overeem Felter, Christian Maare, Annica Margrete Juul Dam, Eirini Tsaggari, Patrick Wohlfahrt

**Affiliations:** a Radiotherapy Research Unit, Department of Oncology, Herlev & Gentofte Hospital, Herlev, Denmark; b Niels Bohr Institute, Copenhagen University, Denmark; c Siemens Healthineers, Forchheim, Germany

**Keywords:** Dual-energy computed tomography, Calibration, Dose calculation, Delineation accuracy, Image guidance

## Abstract

**Background and purpose:**

Dual-energy computed tomography (DECT) is an emerging technology in radiotherapy (RT). Here, we investigate split-filter DECT throughout the RT treatment chain as compared to single-energy CT (SECT).

**Materials and methods:**

DECT scans were acquired with a tin-gold split-filter at 140 kV resulting in a low- and high-energy CT reconstruction (recon). Ten cancer patients (four head-and-neck (HN)​, three rectum​, two anal/pelvis and one abdomen) were DECT scanned without and with iodine administered. A cylindrical and an anthropomorphic HN phantom were scanned with DECT and 120 kV SECT. The DECT images generated were: 120 kV SECT-equivalent (CT_mix_), virtual monoenergetic images (VMIs), iodine map, virtual non-contrast (VNC), effective atomic number (Z_eff_), and relative electron density (ρ_e,w_). The clinical utility of these recons was investigated for calibration, delineation, dose calculation and image-guided RT (IGRT).

**Results:**

A calibration curve for 75 keV VMI had a root-mean-square-error (RMSE) of 34 HU in closest agreement with the RSME of SECT calibration. This correlated with a phantom-based dosimetric agreement to SECT of γ_1%1mm_ > 98%. A 40 keV VMI recon was most promising to improve tumor delineation accuracy with an average evaluation score of 1.6 corresponding to “partial improvement”. The dosimetric impact of iodine was in general < 2%. For this setup, VNC vs. non-contrast CT_mix_ based dose calculations are considered equivalent. SECT- and DECT-based IGRT was in agreement within the setup uncertainty.

**Conclusions:**

DECT-based RT could be a feasible alternative to SECT providing additional recons to support the different steps of the RT workflow.

## 1 Introduction

Performing computed tomography (CT) at two different energies, so-called dual-energy CT (DECT), dates back to the invention of the CT scanner [1], [2]. Mainly due to technical challenges, however, DECT first became clinically available in early 2000, with the detection of pulmonary embolism and urinary stone characterization as often cited applications [3], [4].

Different source-based DECT acquisitions exist such as performing two helical scans at two different energies (typically at 80 and 140 kV) [5], two orthogonal sources simultaneously operating at different energies [6], rapid (<0.5 ms) kV switching of a single source [7], [8], and splitting a single-source beam longitudinally using filtration [4], [9]. The latter technique [10], [11], [12], here termed TwinBeam (TB), is the focus of this study.

DECT applications in radiation therapy (RT) are sparse and have widely focused on improving the accuracy in converting CT numbers into stopping-power ratios in proton therapy [5], [9], [13], [14]. In addition, RT photon based DECT studies have focused on individual applications such as CT calibration [15], metal artifact reduction (MAR) [16], [17], delineation [18], [19], [20] and cone-beam CT (CBCT) based image-guided RT (IGRT) [21]. Here, our aim is to review the clinical usability of TB DECT on a limited heterogeneous patient cohort throughout all main steps of the photon RT chain: CT calibration, delineation, dose calculation, and IGRT.

## 2 Materials and methods

### 2.1 DECT imaging and scanning objects

Both phantoms and patients were included. The TB DECT was acquired at 140 kV with a tin-gold (Sn-Au) split filter in the longitudinal direction on a Siemens Somatom go.Open Pro CT scanner (research license). This generates a high- (Sn140 kV) and low-energy (Au140 kV) CT scan, since the Sn filter leads to a higher attenuation of low-energy photons whereas the Au filter increases the attenuation of high-energy photons due to the k-edge of Au at 81 keV. TB DECT at 140 kV increases the spectral separation as compared to 120 kV [22].

The patients were scanned in TB DE mode without and with iodine contrast (100 ml, 300 mg/ml, Iomeron) administered in the same CT scanning session (CTDI_vol_(32 cm) 7–13 mGy per scan and pitch values 0.3–0.45). For patients with head-and-neck (HN) cancer, bolus tracking at 100 Hounsfield units (HU) in aorta followed by a 35 s delay was applied. For patients with abdominal and pelvic cancer, a 90 s delay after injection was applied. Ten consecutive patients (four HN​, three rectum​, two anal/pelvis, and one abdomen) referred to RT were recruited. Written informed consent was obtained. This strategy was chosen to review an overall bulk effect of TB DECT across tumor sites throughout the RT chain at the expense of better statistics for a more homogenous patient cohort. The patients had both curative (5) and palliative (5) intent.

A cylindrical phantom (model 062MA, Cirs Inc.) with tissue-equivalent inserts of known mass density (ρ), relative electron density (ρ_e,w_), and relative stopping-power ratio (*s*_w_) was included. There were 16 inserts in the range 0.2–1.8 g/cc. Six inserts were outer rim replicas with densities 0.5–1.5 g/cc. Further, an anthropomorphic HN phantom (Max-HD™, IMT Inc.) was included. This phantom in addition received a dose equivalent 120 kV single-energy CT (SECT) (CTDI_vol_(32 cm) ∼ 10 mGy).

**Fig. 1:**
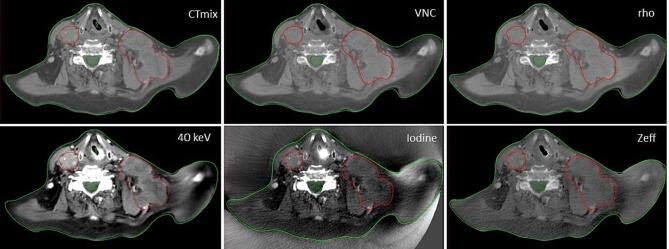
Contrast-enhanced DECT recons for HN patient 2. Top: CT_mix_ (left), VNC (α_w_, middle) and rho (ρ_e,w_, right). Bottom: VMI at 40 keV (left), iodine map (α_I_, middle) and effective atomic number (Z_eff_, right). For the top row and 40 keV, the window (W)/level (L) is 580/50 HU, for iodine W/L is 150/35 HU. Z_eff_ is shown in atomic numbers from 4 to 14. The contours shown are the GTV (red), spinal cord (dark green) and body outline (light green). (For interpretation of the references to colour in this figure legend, the reader is referred to the web version of this article.)

The DECT reconstructions (recons) are described in more detail through equations (S1) - (S4) in the Supplementary material sec. 1. They are: 120 kV SECT-equivalent (CT_mix_), virtual monoenergetic images (VMI), iodine map (α_I_), virtual non-contrast (VNC), effective atomic number (Z_eff_), and relative electron density (ρ_e,w_). All DECT recons were generated in syngo.via (Siemens Healthineers, Forchheim, Germany) from an iterative reconstruction (SAFIRE) using a Qr40 filter. The iBHC filter was off to be consistent with our SECT protocols. ρ_e,w_ is given in HU such that the CT number of air and water are set to −1000 HU and 0 HU, respectively [23]. Fig. 1 shows these recons for a HN patient.

### 2.2 CT calibration and dose calculation

DECT recons of the cylindrical phantom for VMI (60, 70, 75, 78, and 95 keV) and ρ_e,w_ were investigated. For these recons, measured calibration data points (in HU) were generated for the inserts with known ρ, ρ_e,w_ and *s*_w_ to facilitate the different dose calculation algorithms used clinically. The DECT data points were compared to the SECT-based calibration curves in the treatment planning system (TPS, Eclipse v. 16.1, Varian – a Siemens Healthineers company, Palo Alto, CA, USA) using the root mean square error (RMSE) for all inserts including the replicas. The SECT calibration curves consist of preset default values in the TPS [24] for which different SECT protocols (brain, thorax, abdomen etc.) varied 30–40 HU in RMSE. The optimal DECT recon was considered to have a minimum RMSE similar to the SECT RMSE. This strategy was chosen to automatically assign the same calibration curve in the TPS to the same CT scanner regardless of SECT or DECT.

To investigate the dosimetric impact of the DECT RMSE deviations, two gross target volumes (GTVs) and corresponding RT plans in the oral cavity and brain of the HN phantom were created on the SECT scan. The plans were calculated using the AcurosXB_15.6.05 algorithm in the TPS, which utilizes the ρ calibration curve [25]. The oral and brain plans were prescribed 2 Gy and 18 Gy respectively, using a single 6 MV volumetric modulated arc and a 6 MV flattening-filter-free non-coplanar stereotactic radiosurgery (SRS) technique [26] (Varian HyperArc). The plans were re-calculated with fixed settings (MUs, collimator, control points, etc.) on the 60 keV, 75 keV, and ρ_e,w_ DECT recons and compared to the SECT dose distribution using a 3D 1 mm/1% local γ-index with a 10% of Dmax cutoff value [27].

### 2.3 DECT delineation

Approved GTV and organ-at-risk (OAR) contours were transferred to the contrast-enhanced (CE) CT_mix_, VMI (40 and 190 keV), α_I_, VNC, Z_eff_ and ρ_e,w_ recons. Two gastro-intestinal and one HN oncologists were asked to evaluate whether the DECT recons could improve delineation accuracy relative to the CE CT_mix_ on a 4-point Likert scale, with 1 (no improvement), 2 (partly improvement), 3 (improvement) and 4 (great improvement) [28]. This was done by pairwise blending the CT_mix_ (SECT equivalent) and different DECT recons in the TPS providing a score for each contour. Blending was carried out by flipping between the SECT and DECT recon using a graphical slider, i.e. zero percentage mixture of images was expected. In addition, pairwise blending between different DECT recons without the SECT was also evaluated (as compared to the individual SECT vs. DECT). These were 40 vs. 190 keV (40/190 keV), α_I_ vs. VNC (iodine/VNC) and ρ_e,w_ vs. Z_eff_ (rho/Zeff). Window/level settings was made individually. All oncologists evaluated all contours and the scores were pooled according to disease entity (HN, rectum, other and all) and structures (GTVs and OARs). This gave a minimum of 9 evaluations for a pooled group, e.g. the GTV of the 3 rectum patients. Mean values and standard deviations were calculated for each group and significant differences from 1 were identified using a one-tailed one-sample Wilcoxon rank-sum test (p < 0.05) [29].

### 2.4 Dosimetric impact of iodine

The dosimetric consequence of iodine from treatment plans on DECT recons was investigated by comparing non-CE (native) with CE DECT recons for the ten patients. Clinically approved plans optimized on varying DECT recons were re-calculated with fixed settings on the native CT_mix_ and ρ_e,w_, and the CE CT_mix_, VNC and ρ_e,w_. The native and CE CT_mix_ and ρ_e,w_ were used to estimate the impact of iodine in general and on deriving the electron density according to eq. (S4). The VNC was used to remove iodine according to eq. (S1) as compared to a true native recon (see supplementary material sec. 1). All calculations were subject to the same SECT calibration curve in the TPS.

**Fig. 2:**
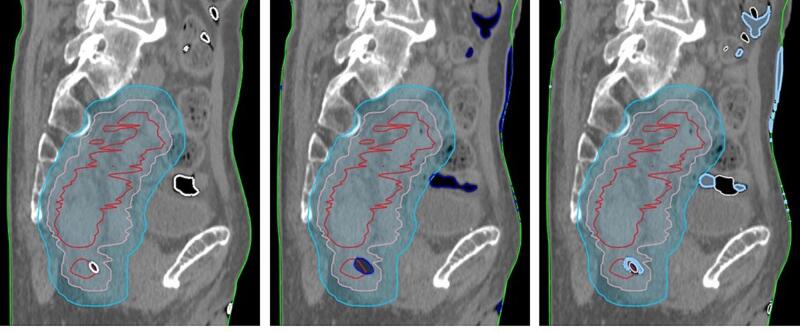
Compensation strategy to reduce the impact of anatomical changes between the native and contrast-enhanced (CE) DECT of a rectum patient with GTV (red), clinical target volume (CTV, pink), PTV (cyan) and body (green) contours. Left: native CT_mix_ with air map (white). Middle: CE CT_mix_ with air map (dark blue). Right: CE CT_mix_ with native air map (white) assigned to air and non-overlapping air map volumes assigned to water (light blue) to reproduce native CT_mix_ anatomical conditions. (For interpretation of the references to colour in this figure legend, the reader is referred to the web version of this article.)

The native CT_mix_ served as a reference. To reduce the impact of changes in the bony anatomy between native and CE scans, all CE recons were rigidly registered to the native CT_mix_ based on the bones. The body contour of the native CT_mix_ was transferred to the CE recons. To reduce the impact of swallowing (HN) and changes in bowel air pockets, the native and CE CT_mix_ air maps were contoured by thresholding (<-300 HU) and the overlap calculated. Non-overlapping volumes of the CE air map were assigned to water (CT number = 0 HU), and the native air map was assigned to air (CT number = -1000 HU) on all CE recons (see Fig. 2). Relevant dose-volume histogram (DVH) points were extracted for comparison. The DVH points describing the planning target volume (PTV) coverage were: the near-maximum absorbed dose (D2%), the near minimum absorbed dose (D98%), and the median absorbed dose (Dmedian) [30]. Two OAR DVH points were, in addition, extracted depending on the anatomical region treated and the contours available.

### 2.5 Dect-based IGRT

**Fig. 3:**
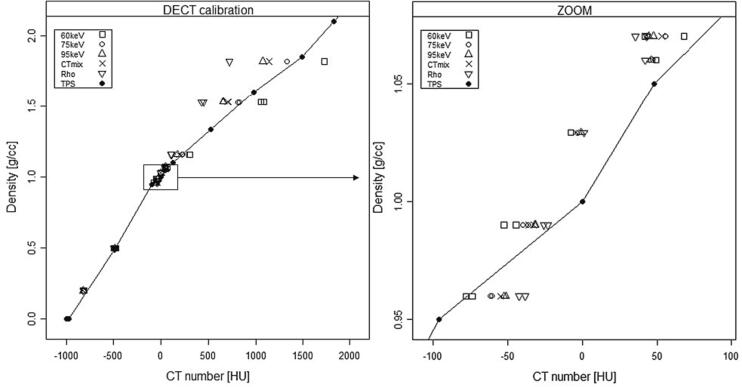
Mass density vs. CT number in HU for different DECT recons as indicated in the figure key (left). The SECT-based calibration curve in the TPS is indicated by closed circles and solid line. The largest spread in CT numbers is seen beyond 1 g/cc. Square insert is a zoom of the soft tissue region (right).

The couch shifts from setup markers to *iso*-center were extracted for the oral and brain plans of the HN phantom. Plans calculated on SECT, ρ_e,w_, VMI at 65 keV and 75 keV were transferred to the linac (Varian TrueBeam) to investigate the impact of DECT image contrast on IGRT accuracy. The rationale for the included image sets was the following: (a) SECT to establish a reference accuracy, (b) 65 keV VMI and ρ_e,w_ having a higher and lower bony contrast as compared to SECT, respectively, and (c) 75 keV VMI being closest to the TPS calibration curve (see Fig. 3). A CBCT with default HN settings (100 kVp, 270 mAs) was acquired from setup and a match (translational 3 degrees of freedom) and subsequent couch shift was applied online based on bony anatomy (200–1700 HU), including the full CBCT field-of-view (lng/lat: 19 cm/26 cm). The couch shift was applied to address any potential quality assurance concerns, e.g. special DICOM tags etc. This procedure was repeated for the two plans and four recons. The online match was compared to the expected couch shift, and the difference calculated. A similar investigation was carried out on the ten patients (see supplementary material sec. 2).

## 3 Results

### 3.1 Calibration and dose calculation

The ρ calibration curves for selected DECT recons are shown in Fig. 3. The CT numbers increase with decreasing energy of VMI as the photoelectric effect becomes more prominent. The ρ_e,w_ recon (in HU) provides an almost linear relationship with ρ. A minimum ρ RMSE of 34 HU was found at 75 keV VMI with similar RMSE of 35 and 28 HU for ρ_e,w_ and *s*_w_, respectively. The γ-index pass rate of the HN plan was 85%, 97% and 98% for the 60 keV VMI, ρ_e,w_ and 75 keV VMI recons. This shows a dependence on RMSE. However, no correlation was observed for the brain plan where all recons had a pass rate > 99%.

### 3.2 Delineation

The results of a DECT-guided delineation accuracy are shown in Table 1. For this limited heterogenous cohort and study protocol, a clear improvement could not be identified with average scores from 1.1 to 1.8 (indicating only partial improvements). The highest gain is seen for the GTV at 40 keV VMI, while no improvements were identified for the OARs using the ρ_e,w_ and Z_eff_ recons.

**Table 1:** Evaluation scores (1–4) of 3 radiation oncologists for the GTV and OARs. Numbers (N) in headers indicate number of patients in the group. Rows indicate the different DECT recons. Every third row, e.g. 40/190 keV is a blend between the two DECT recons. Numbers in cells indicate mean and (standard deviation). Significant differences from 1 (p < 0.05) are shown in bold italic font. OARs HN**: **​spinal cord (4), parotid gland(s) (3), esophagus (2), pharyngeal constrictor (1)​. OARs rectum: bowel (3), bladder (2), sacroiliac (2)​. OAR other (abdomen, anal, pelvis):​ Rectum (2), bladder (1), penile bulb (1), spinal cord (1)​.

	**HN (N = 4)**		**Rectum (N = 3)**		**Other (N = 3)**		**All (N = 10)**	
	*GTV*	*OARs*	*GTV*	*OARs*	*GTV*	*OARs*	*GTV*	*OARs*
**40 keV**	***1.6 (0.9)***	***1.2 (0.4)***	1.4 (0.9)	1.1 (0.4)	***1.6 (0.5)***	1.2 (0.4)	***1.6 (0.8)***	***1.1 (0.4)***
**190 keV**	1.1 (0.3)	1.1 (0.2)	1.0 (0.0)	1.0 (0.0)	1.1 (0.3)	1.0 (0.0)	1.1 (0.3)	1.1 (0.2)
**40/190 keV**	1.3 (0.5)	***1.2 (0.4)***	***1.6 (0.7)***	***1.3 (0.6)***	1.3 (0.7)	1.2 (0.5)	***1.4 (0.6)***	***1.3 (0.5)***
**iodine**	1.2 (0.4)	1.0 (0.2)	1.0 (0.0)	1.0 (0.0)	1.0 (0.0)	1.0 (0.0)	1.1 (0.3)	1.0 (0.1)
**VNC**	1.1 (0.3)	1.0 (0.0)	1.0 (0.0)	1.0 (0.2)	1.0 (0.0)	1.0 (0.0)	1.0 (0.2)	1.0 (0.1)
**iodine/VNC**	***1.8 (1.1)***	***1.2 (0.4)***	1.0 (0.0)	1.0 (0.0)	1.0 (0.0)	1.0 (0.0)	***1.3 (0.7)***	***1.1 (0.3)***
**rho**	1.0 (0.0)	1.0 (0.0)	1.1 (0.3)	1.0 (0.2)	1.0 (0.0)	1.0 (0.0)	1.0 (0.2)	1.0 (0.1)
**Zeff**	1.0 (0.0)	1.0 (0.0)	1.0 (0.0)	1.0 (0.0)	1.0 (0.0)	1.0 (0.0)	1.0 (0.0)	1.0 (0.0)
**rho/Zeff**	***1.4 (0.7)***	1.0 (0.0)	1.0 (0.0)	1.0 (0.0)	1.0 (0.0)	1.0 (0.0)	1.2 (0.5)	1.0 (0.0)

### 3.3 Dosimetric impact of iodine

DVH percentage differences from the native CT_mix_ are shown in Table 2. Differences are expected by residual anatomical changes and the influence of iodine (CE columns), or deviation from the SECT calibration curve (ρ_e,w_ and VNC columns). For this heterogenous patient cohort, there is a good agreement between native CT_mix_ and the different DECT recons, with most differences within 1%. This indicates an overall limited dosimetric impact of iodine on the DECT recons. Differences > 1% are addressed in the discussion.

**Table 2:** Dosimetric deviations of different DECT recons grouped according to anatomical region. Column 1 in gray shows reference DVH points from the native CT_mix_. First 3 sub-columns indicate PTV coverage in % relative to the prescribed dose. Last 2 sub-columns indicate OAR DVH point according to specifications (last column). OAR DVH points were chosen according to the constraints in the applied clinical protocols. Subsequent columns show percentage differences of the different DECT recons relative to CT_mix_. Differences > ±1% are shown in bold italic. Native = non-contrast enhanced, CE = contrast-enhanced, CT_mix_ = 120 kV SECT equivalent, VNC = virtual non-contrast, ρ_e,w_ = relative electron density.

### 3.4 Dect-based image guidance

The deviations of SECT- and DECT-based CBCT matches of the HN phantom from TPS-calculated shifts can be seen in Table 3. All DECT-based match deviations are < 2 mm with no apparent systematic trends. This is further supported by the patient IGRT investigation (see supplementary material sec. 2).

**Table 3:** DECT-based IGRT. Column 1: Translational directions (3 degrees of freedom, DoF) for the brain (top) and oral (bottom) plans. Column 2: Couch shifts from setup markers to *iso*-center calculated in the TPS. Differences (Δ) from TPS shift according to CBCT match with SECT, ρ_e,w_, 75 keV and 60 keV VMI (columns 3–6). SECT-DECT differences (including setup uncertainties) are given in parenthesis (columns 4–6).

**SRS brain**	**TPS calc [mm]**	Δ **SECT [mm]**	Δρ**_e,w_ [mm]**	Δ **75 keV [mm]**	Δ **60 keV [mm]**
**x (lat)**	**−1**	0.5	0.6 (-0.1)	0.5 (0)	0.5 (0)
**y (lng)**	**22**	−2.3	−1.2 (-1.1)	−1.0 (-1.2)	−1.5 (-0.7)
**z (vrt)**	**41**	1.2	1.4 (-0.2)	1.5 (-0.3)	1.5 (-0.3)

**HN oral**					

**x (lat)**	**−5**	0.1	0.6 (-0.5)	0.5 (-0.4)	0.4 (-0.3)
**y (lng)**	**−96**	−0.3	−0.3 (0)	−0.3 (0)	−1.6 (1.3)
**z (vrt)**	**53**	0.9	1.4 (-0.5)	1.2 (-0.3)	1.4 (-0.5)

## 4 Discussion

DECT-based radiotherapy can impact all steps in the RT treatment chain. Therefore, we reviewed the clinical usability of DECT in all main steps of this chain using phantoms and a limited heterogenous patient cohort acquired with one consistent DECT technique.

The DECT calibration curve can be adjusted by weighting the VMI (through eq. (S2), see supplementary material sec. 1) to be aligned with a SECT calibration curve. We acquired TB DECT at 140 kV and found that a VMI at 75 keV had the lowest RMSE similar to a SECT calibration. This provides some practical advantages, especially for larger clinics, because pre-existing scanner-specific SECT calibration curves can continue to be automatically assigned regardless of whether DECT is being used. Alternatively, the DECT DICOM header could be modified with a VMI specific scanner name using e.g. the syngo.via platform before import to the TPS.

A limited number of studies investigate the clinical value of DECT-based delineations. A PubMed database search with the criteria “DECT^1^” and “RT^1^“ and “delineation” resulted in only 12 publications, which mainly focused on optimal DECT recons in terms of maximum contrast or signal-to-noise ratios (CNR/SNR) or MAR considerations [12], [18], [19], [20], [31], [32], [33], [34], [35], [36], [37], [38]. Tumors and OARs in the brain, HN, lung, liver and pancreas were investigated, and in general VMI < 60 keV had favorable CNR as compared to 120 kV SECT. In this study, oncologists evaluated whether existing contours could be improved solely based on different DECT image contrasts. This approach has limitations as many structures were delineated with multimodal imaging available such as magnetic resonance (MRI), positron emission tomography (PET), and journal records. This sometimes meant that a unique relationship between contour improvement and DECT contrast wasn’t feasible. Also, no quantitative metrics such as the dice coefficient could be calculated as contours were not modified. We chose this approach to get an overall gross clinical assessment of DECT-based delineation from three clinical oncologists. Further, this approach reduced distraction from the DECT focus. A significant restriction of the included limited heterogenous patient cohort is a reduction in statistical power of the results. Instead, the results indicate which treatment sites and areas of research that could potentially benefit from further DECT investigation. Given these restrictions, the most promising DECT recons for improving the delineation accuracy seems to be VMI at 40 keV for HN (see e.g. Fig. 1), which is in line with other studies [20]. This seems to be correlated with the average GTV iodine enhancement which was 29 HU (HN), 10 HU (other) and 1 HU (rectum) as quantified in the iodine maps, $\alpha_{I}$. These relatively low enhancement values could explain parts of the lower scoring. Additional explanations could be a lower spectral separation of TB as compared to 80/Sn140 kV DECT (see supplementary material sec. 3). Interestingly, complementary blending such as 40 vs. 190 keV and iodine vs. VNC maps seems to provide a clinical value. The Z_eff_ and ρ_e,w_ recons show no improvement in this setup but could be due to unfavorable window/level settings. Also, a learning curve is expected with new imaging options, and the oncologists had limited prior DECT experience. Further, the TPS (Eclipse) only had grayscale blending available which is in contrast to e.g. the Siemens syngo.via platform [23]. Here, a colored overlay of different DECT recons is possible, potentially improving the evaluation scores. Evaluation in the TPS was chosen as the learning curve of using another software was considered too steep and a potential cause of distraction from the contour scoring.

Contrast-enhanced SECT in general has little influence on a photon-based dose distribution, the difference being in the order of 1–2% [39], [40], [41]. Hence, estimating the dosimetric influence of VNC and ρ_e,w_ recons seems challenging although phantom-based studies have reported notable dosimetric differences [42]. However, comparable clinical volumes of e.g. contrast-enhanced bladder volumes didn’t result in dosimetrically significant differences [43]. In Table 2, deviations from the native CT_mix_ reference dose distribution are expected from residual anatomical changes in the CE scans (Fig. 2) and deviation from the SECT calibration curve (Fig. 3) in addition to the influence of iodine. These are study limitations keeping all parameters but the DECT CT numbers constant. In addition, inspecting dose differences across treatment sites came at the expense of having only 3–4 patients available within each subgroup. Still the combined dosimetric effects seem small. Rectum patient 10 showed the largest deviation for the bladder volume receiving > 22 Gy (V22Gy). Compared to e.g. rectum patient 9 with small dosimetric deviations, a decrease of around 60 HU between the native/CE CT_mix_ and the other DECT recons for the femur heads are similar for the two patients. The only difference was the steepness of the bladder DVH gradient in the relevant dose region. This difference could be a result of planning rather than DECT dependence. For HN patient 6 and 7, OAR differences are caused by small doses being delivered in the reference plan. Relative to the prescribed dose, the differences are < 0.2 percentage points. For HN patient 2 (see Fig. 1), the differences are probably caused by motion-induced (swallowing) mismatch between the Sn140 kV and Au140 kV exposure (bright blurry artifact close to the trachea in VMI and iodine panels of Fig. 1). For patient 4 (abdominal tumor), the dose difference is again caused by a motion artifact in a bowel cavity similar to patient 2. However, all differences are within 2%.

We reviewed the clinical usability of TB DECT throughout the RT treatment chain. A VMI recon at 75 keV works well with SECT-equivalent calibration curves in our clinical setup. Both acceptable CT number and dosimetric deviations as compared to SECT-based RT could be achieved for the included limited heterogenous patient cohort. For HN patients, a VMI at 40 keV seems most promising for improved delineation accuracy. Virtual removal of contrast seems dosimetrically reasonable, although the effect might already be small. DECT-based IGRT is equivalent to SECT within the setup uncertainty for both the phantom and patients included in this study. Overall, DECT-based RT seems to be a feasible alternative to SECT in the different steps of the treatment chain.

## CRediT authorship contribution statement

**Jens Edmund:** Conceptualization, Formal analysis, Funding acquisition, Investigation, Writing – original draft. **Marianne Feen Rønjom:** Investigation, Writing – review & editing. **Mette van Overeem Felter:** Investigation, Writing – review & editing. **Christian Maare:** Investigation, Writing – review & editing. **Annica Margrete Juul Dam:** Investigation, Writing – review & editing. **Eirini Tsaggari:** Investigation, Writing – review & editing. **Patrick Wohlfahrt:** Conceptualization, Funding acquisition, Writing – review & editing.

## Declaration of Competing Interest

The authors declare the following financial interests/personal relationships which may be considered as potential competing interests: Patrick Wohlfahrt is an employee of Siemens Healthineers within the CT research and development section.

## Footnotes

1 Various wording of DECT and RT were used.
